# An Essay on the Shaking Palsy

**Published:** 1817-11

**Authors:** 


*An Essay on the Shaking Palsy.* By JAMES PARKINSON, Member of the Royal College of Surgeons. Sewed, pp. 66. London, 1817.

"THE disease, respecting which the present inquiry is made, is of a nature highly afflictive. Notwithstanding which, it has not yet obtained a place in the classification of Nosologists." Mr. P. has had the opportunity of observing the range of symptoms in this tedious disease for several years, and has hence been led particularly to observe several other cases in which the disease existed in different stages of its progress. By these repeated observations, he thinks he has been led to a probable conjecture as to its true nature and proper mode of treatment, and justly considers it his duty to give publicity to his opinions. Mr. P. needs be under no dread of any "censure which the precipitate publication of mere conjectural suggestions may incur;" his pamphlet does not come under any such title; and the name of the author would be a sufficient passport to publicity, and security from aspersion, for a much less respectable performance.

Chap. I. The term "Shaking Palsy" has been vaguely employed by medical writers. By some it has been restricted to ordinary cases of palsy; by others applied to certain anomalous affections, not belonging to palsy. In the present instance, the agitation produced by the peculiar species of tremor, which here occurs, is chosen to furnish the epithet by which this species of palsy may be distinguished.

History. — So imperceptible is the approach of this malady, that the precise period of its commencement is seldom recollected by the patient. A slight sense of weakness, with a proneness to trembling, sometimes in the head, but most commonly in the hands or arms, are the first symptoms noticed. These affections gradually increase, and, at the period perhaps of twelve months from their first being observed, the patient, particularly while walking, bends himself forward. Soon after this, his legs suffer similar agitations and loss of power with the hands and arms.

As the disease advances, the limbs become less and less capable of executing the dictates of the will, while the unhappy sufferer seldom experiences even a few minutes suspension of the tremulous agitation; and should it be stopped in one limb, by a sudden change of posture, it soon makes its appearance in another. Walking, as it diverts his attention from unpleasant reflections, is a mode of exercise to which the patient is in general very partial. Of this temporary mitigation of suffering, however, he is soon deprived. When he attempts to advance, he is thrown on the toes and fore part of his feet, and impelled, unwillingly, to adopt a running pace; is in danger of falling on his face at every step. In the more advanced stage of the disease, the tremulous motions of the limbs occur during sleep, and augment in violence until they awaken the patient in much agitation and alarm. The power of conveying the food to the mouth is impeded, so that he must submit to be fed by others. The torpid bowels require stimulating medicine to excite them into action Mechanical aid is often necessary to remove the fæces from the rectum. The trunk is permanently bowed. Muscular power diminished. Mastication and deglutition difficult; and the saliva constantly dribbles from the mouth. The agitation now becomes more vehement and constant; and when exhausted nature seizes a small portion of sleep, its violence is such as to shake the whole room. The chin is almost immovably bent down upon the sternum; the power of articulation is lost; the urine and fæces are discharged involuntary; and coma, with slight delirium, closes the scene.

*Case* 1. A gardener, aged 50, remarkably sober and temperate, who had never suffered from rheumatism, pains in the head, or any apoplectic seizure, first noticed a slight trembling of his left hand and arm, after more than ordinary exertion. In the progress of this case, every circumstance mentioned in the preceding history occurred.

*Case* 2. A man, 64 years of age, casually met in the street, had suffered from the disease about eight or ten years. He described it as having come on very gradually, and attributed it to irregularities in living, but particularly to indulgence in spirituous liquors. All the extremities were agitated; the speech interrupted; and the body bowed and shaken. He walked so entirely on the fore part of his feet, that he would have fallen, had it not been for the assistance of a stick.

*Case* 3. The subject of this Case was also met in the street. He was a man of a remarkably athletic frame, 65 years of age. The agitation of the limbs, head, and whole body, was too violent to allow it to be designated as *trembling*. The body was bowed, and the head thrown so forward as to oblige the man to go on a continual run, and employ his stick every five or six steps, to force himself into an upright posture. The man stated he had been a sailor, and thought his complaint arose from lying on the bare earth during a confinement of some months in a Spanish prison.

*Case* 4. A gentleman, ætat. 55, who had experienced a trembling of his arms for about five years, had inflammation of the side, which terminated in an abscess, and discharged a quantity of matter daily for two or three weeks, without affecting any alteration in his original complaint.

*Case* 5. A gentleman was seen at a distance supported by an attendant, who was standing before him with a hand on each shoulder. By gently swaying backwards and forwards, he placed himself in equipoise; and, giving the word, started in a running pace, the attendant sliding before him, ready to receive him and prevent his falling after having run about twenty paces.

*Case* 6. A gentleman, ætat. 72, has suffered from the disease some years. Twelve months since, he had hemiplegia of the right side, which was entirely removed in a fortnight, by purgatives, and a blister behind the neck. At present, he is almost constantly troubled with the agitation, but is generally able to stop it for a short period by a sudden change of posture, such, for instance, as throwing himself into a chair. His sleep is much disturbed; he is scarcely able to feed himself; and when he walks, it is with the constant apprehension of falling forwards.

The author is of opinion, that the preceding are all cases of the same disease, differing only in stage and duration.

Chap. II. The pathognomonic symptoms of this malady, which have been considered by Nosologists as constituting two distinct diseases, under the names of "Tremor Coactus" and "Scelotyrbe Festinans," are, 1st. "Involuntary tremulous motion, with lessened voluntary muscular power in parts, not in action, and even supported." 2d. "A propensity to bend the trunk forwards, and to pass from a walking to a running pace."

The disease under consideration differs from tremor, by the agitation occurring whilst the affected parts are supported and unemployed; in the latter, it takes place only on their being put in action. The morbid motions which constitute this disease may be characterised by the term "palpitation."

Chap. III. It is of importance to mark the diagnostic characters of shaking palsy. In palsy consequent on compression of the brain, the diminution of voluntary muscular action is sudden, and the sense of feeling in the affected member is impaired. But the approach of this disease is with extreme slowness, and the parts suffer no diminution of sensibility.

Anomalous convulsive affections have been improperly designated "shaking palsy".—Two cases, one related by

Dr. Charlton, in which an involuntary swinging of the arm, alternated with pain in the stomach and convulsive fits; and the other, that of a boy, the agitation of whose limbs evidently arose from the presence of worms in the primæ viæ, have been cited by Dr. Kirkland, in his Commentary on Apoplectic and Paralytic Affections, as belonging to the class of "shaking palsies."

This disease may be distinguished from the trembling consequent to old age, indulgence in spirituous liquors, or any of those causes which diminish nervous energy, by attending to what has been already said respecting tremor, in the second chapter.

Chap. IV. As the light which anatomical examination would afford has not been obtained, the proximate cause of this disease can only be conjectured. It is, therefore, *supposed* to be a diseased state of the medulla spinalis, originating in that part which is contained within the canal of the cervical vertebræ, and, as the disease proceeds, extending to the medulla oblongata.

The remote causes may be injuries of the medulla or theca vertebralis, causing inflammation and consequent derangement of structure in the medulla itself, and thickening, effusion, or ulceration in its investing membranes. Cases illustrative of this malady are rare.—A. B. ætat. 26, the next morning after exposure to cold, whilst under a course of mercury for a venereal affection, complained of extreme pain in the back, and total inability to voluntary motion in the lower extremities, which were perpetually agitated with palpitatory motions. No advantage was derived from a course of medicines continued twelve months. Ten years afterwards, this patient was met in the street, shifting himself along, seated in a chair. The convulsive motion had ceased, the limbs being totally inert. Between that kind of palsy of the lower limbs accompanying curvature of the spine, and the present disease, no instructive analogy can be discovered. There are, indeed, sometimes, slight convulsive motions of the extremities in the former; but they are different to the palpitations of the latter.

The case of the Count de Lordat, related by Dr. Maty, in the third vol. of Med. Observ. and Inquir. accords, in many symptoms, with the cases already given; and although it may not mark an identity of disease, yet shows that nearly the same parts were affected in both instances. The Count, in consequence of a fall, which bent his head violently from left to right, felt a weakness, first in the left and afterwards in the right arm. Difficulty in articulation, chewing, swallowing, and retaining the saliva, with convulsive motions all over the body, followed in the order they are mentioned, whilst the intellects continued unimpaired to the last.

*Dissection.* Pia mater "full of blood and lymph," having several hydatids on it, and towards the falx marks of suppuration. Ventricles filled with water; plexus choroides gorged with blood, and enlarged. Surface of the brain browner than usual; medullary substance and cerebellum healthy. Medulla oblongata increased more than one third in size. The membranes inclosing the spinal marrow so tough as to be cut with difficulty. Medulla spinalis of so firm a structure as to elude the pressure of the finger: this hardness lessened by degrees, and in the vertebræ of the thorax, the medulla resumed its healthy appearance.

In two of the cases already noticed, symptoms of rheumatism had previously existed. In case four, the right arm, in which the palpitation began, was violently affected with rheumatic pains to the fingers' ends. This led to the supposition that the latter circumstance might be the cause of the palpitations and other symptoms, and influenced the treatment adopted in the following cases.

A. B. subject to rheumatic affections of the deltoid muscle, felt intense pain extending down the arm, along the inside of the fore-arm to the sides of the fingers, in which a tingling was felt. There was pain also on the fore and back parts of the chest, augmented by a full inspiration. The course of the pain being that pursued by the brachial nerve, suggested the probability of increased determination to its origin and the neighbouring medulla. Cupping, hot fomentations, and a blister to the back of the neck, with small doses of antimonials and a purge of calomel, removed the pain entirely in four or five days. A painful weariness of the arm after exertion was present for some time after, but no palpitation or any other unpleasant symptom.

A female, aged 40, complained of great pain in both the arms, extending from the shoulder to the fingers' ends. She had a similar attack nine months before, which was considered as rheumatism, but not benefited by the usual medicines: after three or four weeks, however, it gradually amended, leaving her arms and hands in a weakened and trembling state. A similar plan of treatment to that adopted in the preceding case was followed by entire removal of pain. Farther information as to the progress of the case could not be obtained.

On meeting with these two cases, it was thought probable that rheumatic affections, laid the foundation of this disease, which might manifest itself at a distant period, when the circumstance in which it originated might be forgotten.

Chap. V. It is assumed, that the disease depends on a disordered state of that part of the medulla contained within the cervical vertebræ. The treatment best calculated to afford relief appears to be, the abstraction of blood from the upper and back part of the neck, followed by vesicatories, kept discharging by the Sabine liniment; or the formation of caustic issues on each side the cervical vertebræ. It should be remembered that; "As slow as is the progress of the disease, so slow, in all probability, will be the return to health." These means, therefore, must not be hastily abandoned.

Until better information is obtained respecting the nature of the disease, internal medicines hold out no rational hope for remedy; but, from its well known influence in correcting derangement of structure, the trial of mercury will not be neglected by the judicious.

Care should be taken not to mistake the weakness of the muscles in this disease, for the mere consequence of constitutional debility, as the tonic medicines and nutritious diet so beneficial in the latter, would prove highly injurious in the former complaint. The power of sympathy in inducing disorder of function, and even derangement of structure, in a distant part, cannot be denied. In the following case, that sympathetic influence which so closely simulates the forms of other diseases, induced symptoms greatly resembling those of this malady.

A. B. ætat. 54, of temperate habits and regular bowels, became gradually affected with numbness, pricking, and weakness of his arms, with a sense of fulness about the shoulders; and at times trembling of the hands. The appetite had diminished for several weeks; and the abdomen felt as though containing considerable accumulation. On emptying the bowels with calomel and Epsom salts occasionally, the complaints were entirely removed.

" Before concluding these pages, it may be proper to observe once more, that an important object proposed to be obtained by them is, the leading of the attention of those who humanely employ anatomical examination in detecting the causes and nature of diseases, particularly to this malady. Little is the public aware of the obligations it owes to those who, led by professional ardour, and the dictates of duty, have devoted themselves to these pursuits, under circumstances most unpleasant and forbidding. Every person of consideration and feeling, may judge of the advantages yielded by the philanthropic exertions of a Howard; but how few can estimate the benefits bestowed on mankind, by the labours of a Morgagni, Hunter, or Baillie."

The reader will now perceive, that this little pamphlet is highly worthy of perusal, and deserves the attention of the medical public.

